# Sterols and Terpenoids from *Viburnum odoratissimum*

**DOI:** 10.1007/s13659-014-0021-7

**Published:** 2014-05-14

**Authors:** Jun-Zeng Ma, Xing-Wei Yang, Jing-Jing Zhang, Xia Liu, Li-Lan Deng, Xiao-Ling Shen, Gang Xu

**Affiliations:** 1 Southwest Forestry University, Kunming, 650224 People’s Republic of China; 2 State Key Laboratory of Phytochemistry and Plant Resources in West China, Kunming Institute of Botany, Chinese Academy of Sciences, Kunming, 650201 People’s Republic of China; 3 Laboratory of Chinese Herbal Drug Discovery, Tropical Medicine Institute, Guangzhou University of Chinese Medicine, Guangzhou, 510405 People’s Republic of China

**Keywords:** *Viburnum odoratissimum*, Insulin sensitizing activity, 6*α*-Hydroxy-lup-20(29)-en-3-on-28-oic acid, Viburodorol A

## Abstract

**Abstract:**

A new stigmasterol type natural product, viburodorol A (**1**), along with eleven known sterols and terpenoids (**2**–**12**), were isolated from the aerial parts of *Viburnum odoratissimum.* The structure of **1** was elucidated on the basis of comprehensive spectroscopic analysis. It’s noteworthy that compound **2**, the major constituent of this plant, can significantly stimulate glucose absorption in insulin resistant HepG2 cells without affecting cell viability. Furthermore, this compound can also restore the glucose absorption in DXMS-induced insulin resistant 3T3-L1 cells.

**Graphical Abstract:**

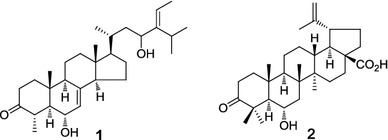

**Electronic supplementary material:**

The online version of this article (doi:10.1007/s13659-014-0021-7) contains supplementary material, which is available to authorized users.

## Introduction

Diabetes mellitus (DM) is a heterogeneous group of disorders characterized by hyperglycemia [1], resulting from absolutely insulin deficiency, insulin resistant and/or abnormal secretion [2]. Over 90 % of diabetes cases around the world belong to Type 2 diabetes [3], a heterogeneous, polygenic disorder resulting from interaction between susceptibility genes and lifestyle/environmental factors [4]. About 4.8 million people died and 471 billion USD were spent due to DM in 2012 globally. According to a latest survey, the number of people living with DM has reached up to 371 million; unfortunately, the number is keep on rising [5]. DM may lead to a series of complications, such as blindness, renal failure, nerve damage, stroke, and limb amputation [6]. Currently available therapies for DM include some main classes of agents such as sulphonylureas, biguanides, *α*-glucosidase inhibitors, and thiazolidinediones [7]. However, they may show different kinds of adverse effects in varying degrees, like gastrointestinal disturbances, and hypoglycemia [4]. Thus, more effective and safer agents need to be found.

Insulin resistance is the main character of type 2 diabetes which leads to decreased glucose absorption by fat, liver, skeleton muscle, etc. A potential anti-diabetic drug should be highly active in restoring glucose absorption and none or less toxic to body tissues. Recently, we found that the extract of *Viburnum odoratissimum* significantly stimulated glucose absorption in insulin resistant HepG2 cells at concentrations that did not affect cell viability. At the concentration of 25 μg/mL, the extract increased glucose consumption of insulin resistant HepG2 cells by 7.5 %, showing ability to stimulate glucose absorption and restore insulin sensitivity of insulin resistant HepG2 cells.

**Fig. 1:**
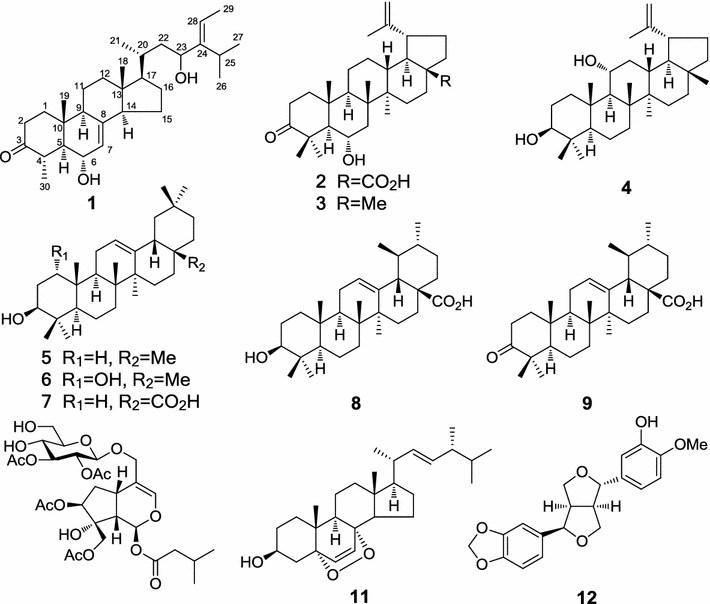
Structures of compounds **1–12**

*V. Odoratissimum* Ker-Gawl was an evergreen shrub or dungarunga distributed in southeast of China and used in folk as a medicine to diminish inflammation, relieve pain, and treat rheumatism [8]. Several diterpenes, triterpenes, flavones, lignans, and coumarins have been reported as the main secondary metabolites of this plant [9]. With the aim of searching for structural interesting metabolites with insulin sensitizing activities, the chemical constituents of this plant was investigated, and a new stigmasterol type natural product, viburodorol A (**1**), along with eleven known sterols and terpenoids (**2**–**12**), were isolated (Fig. 1). It’s noteworthy that compound **2**, the major constituent of this plant in this study, can also significantly stimulate glucose absorption in insulin resistant HepG2 cells without affecting cell viability. Furthermore, this compound can also restore the glucose absorption in DXMS-induced insulin resistant 3T3-L1 cells. Reported herein, was the isolation and structural elucidation of these natural products as well as insulin sensitizing activity of **2**.

## Results and Discussion

The methanol extract of the air-dried and powdered aerial parts of *V. odoratissimum* (4.0 kg) was subjected to a silica gel column to afford five fractions A–E. Fractions B, C and D were subjected to a series of chromatographic methods, and led to the isolation of a new steroid (viburodorol A, **1**) together with 11 known compounds 6*α*-hydroxy-lup-20(29)-en-3-on-28-oic acid (**2**) [10], rigidenol (**3**) [11], nepeticin (**4**) [12], *β*-amyrin (**5**) [13], castanopsol (**6**) [14], oleanolic acid (**7**) [15], ursolic acid (**8**) [16], ursonic acid (**9**) [17], 7,10,2′,3′tetra-acetylsuspensolide F (**10**) [18], 5*α*,8*α*-epidioxy-24-norcholesta-6,22-dien-3*β*-ol (**11**) [19], and horsfieldin (**12**) [20], respectively. In the bioassay for insulin sensitizing activities of the isolates, compound **2** significantly stimulated glucose uptake in insulin resistant HepG2 cells without affecting cell viability, showing ability to restore the impaired insulin sensitivity of cells.

Compound **1** was obtained as white powder. Its molecular formula, C_30_H_48_O_3_, was established by HRTOFMS (*m/z* 479.3504, [M + Na]^+^, calcd 479.3501), indicating 7 indices of hydrogen deficiency. The IR spectrum showed obvious absorption bands for hydroxyl (3416 cm^−1^), olefinic (2957 cm^−1^), and carbonyl groups (1711 cm^−1^). The ^1^H-NMR spectrum (Table 1) indicated clearly the presence of seven methyls including five doublets (*δ*_H_ 1.15, *J* = 6.4 Hz; 1.19, *J* = 7.1 Hz; 1.21, *J* = 7.1 Hz; 1.71, *J* = 7.0 Hz and 1.74, *J* = 6.7 Hz) and two singlets (*δ*_H_ 0.61 and 1.04). The ^13^C-NMR and DEPT spectral data (Table 1) displayed 30 carbon resonances including seven methyls, seven aliphatic methylenes, eleven methines with two olefinic (*δ*_C_ 125.1 and 117.7) and two oxygenated (*δ*_C_ 71.1 and 69.6), and five quaternary carbons including two olefinic (*δ*_C_ 138.8 and 150.7) and one carbonyl group at *δ*_C_ 213.3. Considering the characteristic signals for seven methines at C-4 (*δ*_H_ 2.75, *δ*_C_ 46.7), C-5 (*δ*_H_ 1.64, *δ*_C_ 57.4), C-9 (*δ*_H_ 1.78, *δ*_C_ 48.6), C-14 (*δ*_H_ 1.85, *δ*_C_ 55.0), C-17 (*δ*_H_ 1.23, *δ*_C_ 57.2), C-20 (*δ*_H_ 2.07, *δ*_C_ 33.7), and C-25 (*δ*_H_ 2.86, *δ*_C_ 28.5), two quaternary signals for C-10 (*δ*_C_ 37.1) and C-13 (*δ*_C_ 43.8), together with the seven typical methyls mentioned above, **1** could be ascribed to be a steroid type metabolite related closely to stigmasterol with thirty carbons in the skeleton [21, 22].

**Table 1:** ^1^H and ^13^C NMR data for compound **1** (in C_5_D_5_N, *δ* in ppm, *J* in Hz)^a^

Position	*δ*_C_	*δ*_H_ (*J* in Hz)	Position	*δ*_C_	*δ*_H_ (*J* in Hz)
1	37.9, t	1.91, m	15	23.3, t	1.50, m
		1.43, m			1.44, m
2	37.3, t	2.47, dt (4.8, 11.6)	16	28.4, t	1.91, m
		2.39, m			1.46, m
3	213.3, s		17	57.2, d	1.23, m
4	46.7, d	2.75, m	18	12.3, q	0.61, s
5	57.4, d	1.64, t (9.6)	19	14.5, q	1.04, s
6	71.1, d	4.30, d (7.6)	20	33.7, d	2.07, br. s
7	125.1, d	5.50, s	21	19.0, q	1.15, d (6.4)
8	138.8, s		22	45.3, t	1.93, m
9	48.6, d	1.78, m			1.23, m
10	37.1, s		23	69.6, d	4.48, d (10.4)
11	21.6, t	1.52, m	24	150.7, s	
		1.41, m	25	28.5, d	2.86, sept (7.1)
12	39.6, t	2.13, m	26	21.8, q	1.19, d (7.1)
		1.23, m	27	21.7, q	1.21, d (7.1)
13	43.8, s		28	117.7, d	5.87, q (7.0)
14	55.0, d	1.85, m	29	13.5, q	1.71, d (7.0)
			30	16.7, q	1.74, d (6.7)

^a^600 MHz for ^1^H and 150 MHz for ^13^C NMR experiments, respectively

**Fig. 2:**
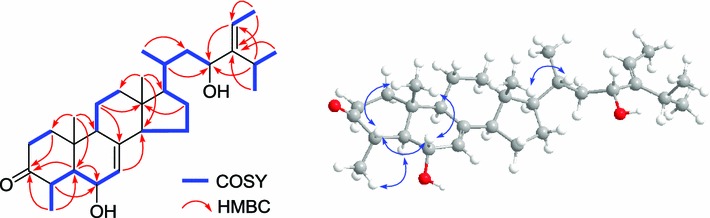
Key ^1^H-^1^H COSY(), ^1^H-^13^C HMBC (), and ^1^H-^1^H ROESY () correlations for **1**

Detailed comparison of the NMR spectral data of **1** with those of 4*α*-methyl-24*S*-ethyl-5*α*-cholestan-3*β*-ol indicated that they are similar to one another [21]. Comparatively, the oxygenated methine at C-3 of the known analogue was replaced by a carbonyl group (*δ*_C_ 213.3) in **1**, which can be confirmed by its HMBC correlations with H-1 (*δ*_H_ 1.91), H-5 (*δ*_H_ 1.64) and H-30 (*δ*_H_ 1.74). In addition, C-6 (*δ*_C_ 71.1) and C-23 (*δ*_C_ 69.6) were both substituted by a hydroxyl group, as supported by HMBC correlations from H-4 (*δ*_H_ 2.75) and H-5 (*δ*_H_ 1.64) to C-6 (*δ*_C_ 71.1), together with H-20 (*δ*_H_ 2.07) and H-25 (*δ*_H_ 2.86) to C-23 (*δ*_C_ 69.6), respectively. The two pairs of double bonds in **1** can be ascribed to C-7/C-8 and C-24/C-28, suggested by HMBC correlations from H-5 (*δ*_H_ 1.64) to C-7 (*δ*_C_ 125.1), H-11 (*δ*_H_ 1.52) to C-8 (*δ*_C_ 138.8), and from H-25 (*δ*_H_ 2.86) and H-29 (*δ*_H_ 1.71) to C-28 (*δ*_C_ 117.7). Thus, the planar structure of **1** was elucidated and can be confirmed by the undoubted HMBC correlations of the seven methyls (Fig. 2), as well as the six proton systems obtained by ^1^H-^1^H COSY experiment, H-1/H-2, Me-30/H-4/H-5/H-6/H-7, H-9/H-11/H-12, H-14/H-15/H-16/H-17/H-20/Me-21/H-22/H-23, Me-26/H-25/Me-27, and Me-29/H-28.

The relative configuration of **1** was deduced based on ROESY experiment conjugated with the biogenetic analysis. Biogenetically, Me-18 and Me-19 are both *β*-oriented, while H-5, H-9, H-14, and H-17 are *α*-oriented. The ROESY cross-peaks of H-4/Me-19, H-4/H-6, H-6/Me-19, and H-5/Me-30 suggested that H-4 and H-6 were both *β*-oriented. Cross-peaks of Me-18/H-20 implying that H-20 was *β*-orientation. In addition, the configuration of C-23 can not be defined. Therefore, the structure of **1** was deduced as shown and named viburodorol A.

In addition, insulin resistance in HepG2 cells was induced by dexamethasone (DXMS) treatment and the new (**1**) and major constituent (**2**) were assessed the activity in enhancing glucose consumption of the cells. The result exhibited that compound **2** can significantly stimulate glucose uptake in insulin resistant HepG2 cells (P < 0.05–0.001, Table 2) without affecting cell viability, showing ability to restore the impaired insulin sensitivity of cells. At the concentrations of 10, 5, 2.5, 1.25 μmol/L, compound **2** increased glucose consumptions of insulin resistant HepG2 cells from 71.6 % to 168.7, 155.9, 128.3 and 90.1 % respectively, showing excellent activity in stimulating glucose uptake. Further investigation on the activity in murine fat cells of **2** was also carried out. DXMS was also employed for induction of insulin resistant as described above. Rosiglitazone which is a typical insulin sensitizer was used as positive drug control. Table 3 showed that, unlike rosiglitazone which strongly prompted glucose uptake in both insulin sensitive and resistant 3T3-L1 adipocytes, (P < 0.001 and 0.01), Compound **2** at 10 or 5 μmol/L did not obviously increase glucose consumption in sensitive cells (P > 0.05) but completely restored the impaired glucose uptake in DXMS-induced insulin resistant cells (P < 0.05). These results suggested that **2** might be a promising candidate as insulin sensitizer for treatment of type 2 diabetes.

**Table 2:** Effect of **2** on glucose consumption and viability of DXMS-induced insulin resistant HepG2 cells

Sample	C_sample_ (μmol/L)	Relative glucose consumption (%)	Cell viability (%)
**2**	10	168.7 ± 6.8***	87.8 ± 2.3
	5	155.9 ± 9.2***	88.9 ± 1.7
	2.5	128.3 ± 14.0**	92.6 ± 2.6
	1.25	90.1 ± 9.4	91.4 ± 2.7
Insulin resistant control		76.1 ± 12.7^▲^	92.0 ± 5.5
Insulin sensitive control		100	100

^▲^*P* < 0.05 as compared to insulin sensitive control

*P < 0.05, **P < 0.01 and **P < 0.001 as compared to insulin resistant control

**Table 3:** Effect of **2** on glucose consumption in insulin resistant and sensitive 3T3-L1 adipocytes

Treatment (μmol/L)		Relative glucose consumption (%)		Cell viability (%)
		Sensitive (without DXMS)	Resistant (with DXMS)	
Control		100	84.6 ± 4.5^▲^	100
**2**	10	105.8 ± 4.1	102.2 ± 2.9^**^	99.1 ± 3.0
	5	105.5 ± 4.3	100.6 ± 1.9^*^	100.0 ± 4.6
	1	102.8 ± 2.9	85.8 ± 4.2	99.9 ± 1.0
Ros	20	123.2 ± 3.1^▲▲▲^	111.6 ± 3.8^***,▲▲^	102 ± 4.2

Ros Rosiglitazone

^▲ ^P < 0.05, ^▲▲ ^P < 0.01 and ^▲▲▲ ^P < 0.001 as compared with insulin sensitive control

*P < 0.05, **P < 0.01 and ***P < 0.001 as compared with insulin resistant control

## Experimental Section

### General Experimental Procedures

Optical rotations were measured with a HORIBA SEPA-300 high-sensitive polarimeter. IR spectra were determined on a Bruker Tensor-27 infrared spectrophotometer with KBr disks. UV spectra were recorded on a Shimadzu UV2401A ultraviolet–visible spectrophotometer. HRTOFMS analysis was carried out on an API QSTAR time-of-flight spectrometer and on a Waters Auto Spec Premier P776 mass spectrometer. NMR spectra were recorded on Bruker AV400, DRX-500 and DRX-600 spectrometers at 25 °C, using TMS as an internal standard. Chemical shifts were reported in units of *δ* (ppm) and coupling constants (*J*) were expressed in Hz. Column chromatography (CC) were carried out over silica gel (200–300 mesh, Qingdao Haiyang Chemical Co. Ltd), Rp-18 (40–63 μm, Merck), and Sephadex LH-20. Pre-coated silica gel plates (Qingdao Haiyang Chemical Co. Ltd.) were used for TLC. Detection was done under UV light (254 and 365 nm) and by spraying the plates with 10 % sulfuric acid followed by heating. An Agilent series 1100 (Agilent Technologies) were used for HPLC. An Agilent ZORBAX SB-C_18_ column 5 μm 143 Å column (250 × 9.4 mm) were used for semi-preparative HPLC separations.

### Plant Material

The aerial parts of *V. odoratissimum* were collected in Kunming Botany Garden, Yunnan Province, P. R. China, in October 2008. The plant was identified by Dr. Xiao Cheng, Kunming Institute of Botany, Kunming, P. R. China. A voucher specimen was deposited at Kunming Institute of Botany with identification number 200810V02.

### Extraction and Isolation

The air dried stems and leaves of the plant (4 kg) were pulverized and extracted with MeOH(30 L × 3, each time 2 day). The residue (268 g) was subjected to silica gel CC eluting with PE/EtOAc (1:0–0:1) to give a total of five fractions, A–E. Fraction B was subjected to a silica gel CC using (PE/Acetone, 1:0–0:1) to obtain four fractions, B1–B4. After purification over Reverse Phase carbon-18 (RP-18, MeOH/H_2_O, 45–80 %) and silica gel CC (PE-CHCl_3_-EtOAc), fraction B2 afforded **4** (3 mg), **11** (9 mg), **9** (5 mg). Fraction B3 was separated over RP-18 (MeOH/H_2_O, 45–80 %) and Sephadex LH-20 (CHCl_3_/MeOH, 1:1) to give **1** (30 mg), **2** (565 mg), and **3** (2 mg). Fraction C was subjected to CC on silica gel (PE/EtOAc, 1:0–0:1) to obtain three fractions, C1–C3. After purification over RP-18, followed by Sephadex LH-20 (CHCl_3_/MeOH, 1:1), fraction C2 afforded **7** (4 mg) and **8** (3 mg). Fraction D was separated over RP-18 (MeOH/H_2_O, 20–100 %) into fractions D1–D4. After purification by silica gel CC (PE/EtOAc, 1:0–0:1) and Sephadex LH-20 (CHCl_3_/MeOH, 1:1) together with semi-preparative HPLC, fraction D1 afforded **6** (8 mg), **12** (2 mg), **5** (4 mg), and **10** (3 mg).

#### Viburodorol (**1**)

White amorphous powder; $[\alpha ]_{\text{D}}^{24}$ + 40.0 (*c* 0. 2, CHCl_3_); UV (CHCl_3_) λ_max_ nm (log *ε*): 191 (2.33); IR (KBr) *ν*_max_: 3416, 2957, 2870, 1711, 1639, 1461, 1381, 1101, 1025 cm^−1^; ^1^H and ^13^C NMR data: see Table 1; HRTOFMS: *m/z* 479.3504 [M + Ma]^+^; calcd for C_30_H_48_O_3_Na, *m/z* 479.3501.

### Insulin Sensitizing Activity

#### General

Dulbecco’s modified eagle medium (DMEM) and fetal bovine serum (FBS) were purchased from Gibco (Shanghai, China). Glucose assay kits were purchased from Changchun Huili Biotech Co., Ltd (Changchun, China). Cell counting kit-8 (CCK-8) was provided by Dojindo Laboratorise (Shanghai). Dexamethasone (DXMS), insulin and DMSO were purchased from Sigma (Shanghai, China).

#### Cell Line and Cell Culture

Human hepatoma cell line HepG2 was purchased from the Laboratory Animal Center of Sun Yat-Sen University (Guangzhou, China) and was maintained in DMEM containing 2.0 g/L of glucose and 10 % of FBS. Murine preadipocyte cell line 3T3-L1 was a gift from Prof. WF Fong of Hong Kong Baptist University and was maintained in DMEM containing 4.5 g/L of glucose and 10 % of FBS. Both cell lines were incubated at 37 ºC, 5 % CO_2_, and saturate humidity.

#### Glucose Consumption in HepG2 Cells

Effect of test samples on glucose absorption in insulin resistant HepG2 cells were performed in 96-well format. In brief, 8 × 10^3^ HepG2 cells suspended in 100 μL of growth medium (DMEM + 10 % FBS, Gibco products) were seeded in 96-well plates and incubated for 24 h to let cells to adhere to the well bottom. Then the cells were replaced the medium with 100 μL of fresh growth medium containing 1 μmol/L of DXMS and different concentrations of test samples. After 48 h incubation, 5 μL per well of the medium was taken for measurement of glucose concentration using Glucose Assay kits (Changchun Huili Biotech Co., Ltd, China). Cells treated with 1 μmol/L of DXMS alone were set as insulin resistant control while cells without any drug treatment were set as insulin sensitive control, respectively. Glucose consumed by cells of different treatments was expressed as relative glucose consumption (%) over to insulin sensitive control cells whose relative glucose consumption was set as 100 % [23].

#### Glucose Consumption in 3T3-L1 Adipocytes

5 × 10^3^ 3T3-L1 preadipocytes suspended in 100 μL of growth medium were seeded in 96-well plates and let cells grow to confluence. Then cells were induced for adipocyte differentiation in growth medium containing 10 µg/mL of insulin (Sigma-Aldrich), 1 µmol/L of DXMS (Sigma-Aldrich), and 0.5 mmol/L of 3-isobutyl-1-methylxanthine (Sigma-Aldrich) for 48 h, followed in growth medium containing 10 µg/mL of insulin for 48 h, and lastly in fresh growth medium. The induction lasted for 8 days and the media were refreshed every 2 days.

The differentiated adipocytes were replaced the medium with 100 μL fresh growth medium containing different concentrations of test samples. Glucose left in medium after 48 h of incubation was measured by Glucose Assay kits as described above. To investigate the action of test samples on insulin resistant adipocytes, 1 μmol/L of DXMS was added to the medium together with the test sample [24].

#### Cell Viability Assay

After taking 5 μL per well of the medium for glucose consumption assay, the 96-well plate was added 10 μL of CCK-8 (DOJINDO LABORATORISE, Japan) and further incubated at 37 °C for 1.5 h. Optical density (OD) positively correlated with the number of living cells was read at 450 nm. Effects of test samples on cell viability were evaluated according to OD value [25].

#### Statistical Analysis

Relative glucose consumption after 48 h of incubation was expressed as Mean value ± Standard deviation. Statistical analyses were performed by One-way ANOVA. Differences were considered significant when the P value was less than 0.05.

## Electronic supplementary material

Below is the link to the electronic supplementary material.

Supplementary material 1 (DOC 5871 kb)

## Abbreviations

DM: Diabetes mellitus
DXMS: Dexamethasone
OD: Optical density

## References

[1] Bell GI, Polonsky KS (2001). Nature.

[2] Paul Z, KGMM A, Jonathan S (2001). Nature.

[3] World Health Organization, Diabetes/media centre/fact sheet no. 312. (updated March, 2013)

[4] Goldstein BJ, Müller-wieland D (2008). Type 2 Diabetes: Principles and Practice.

[5] International Diabetes Federation (2012). Diabetes Atlas.

[6] Brownlee M (2001). Nature.

[7] Andrew JK, Clifford JB (2005). Drugs.

[8] Su JC (1983). Acta Bot. Sin..

[9] Liu J, Zhou WB, Con YW, Liu P (2013). Acta Pharm. Sin..

[10] Kuroyanagi M, Shiotsu M, Ebihara T, Kawai H, Ueno A, Fukushima S (1986). Chem. Pharm. Bull..

[11] Dantanarayana AP, Kumar NS, Muthukuda PM, Wazeer MIM (1982). Phytochemistry.

[12] Ahmad VU, Bano S, Voelter W, Fuchs W (1981). Tetrahedron Lett..

[13] Brieskorn CH, Kapadia Z (1980). Planta Med..

[14] Murty YLN, Jairaj MA, Sree A (1989). Phytochemistry.

[15] Usmanghani K, Nazir T, Ahmad VU (1982). Fitoterapia.

[16] Bashir AK, Turner TD, Ross MS (1982). Fitoterapia.

[17] Mukherjee KS, Bhattacharya MK, Ghosh PK (1982). Phytochemistry.

[18] Lamberto T, Sebastiano F, Marcello N, Francesca CM, Giovanna P, Corrado G (1997). Phytochemistry.

[19] Yue JM, Chen SN, Lin ZW, Sun HD (2001). Phytochemistry.

[20] Gunatilaka AAL, Jasmin De Silva AMY, Sotheesawaran S, Tillekeratne LMV (1982). Phytochemistry.

[21] Kokke WCMC, Fenical W, Djerassi C (1981). Phytochemistry.

[22] Bohlin L, Kokke WCMC, Fenical W, Djerassi C (1981). Phytochemistry.

[23] Chen HY, Li XG, Ye XL, Jin YN, Huang J, Wu JF (2012). China J. Chin. Mater. Med..

[24] Reed BC, Daniel Lane M (1980). Proc. Natl. Acad. Sci. U. S. A..

[25] Jiang Y, Ahn EY, Ryu SH, Kim DK, Park JS, Yoon HJ, You S, Lee BJ, Lee DS, Jung JH (2004). BMC Cancer.

