# Supplementary material for: Sterols and Terpenoids from Viburnum odoratissimum
Source: Nat Prod Bioprospect. 2014 May 14;4(3):175–80. doi: 10.1007/s13659-014-0021-7 (PMC4050308; doi:10.1007/s13659-014-0021-7)

**Natural Products and Bioprospecting**

**S1** Supplementary data contents page

**Title:** Sterols and Terpenoids from *Viburnum odoratissimum*

**Authors:** Jun-Zeng Ma,*^a,b^ Xing-Wei Yang,^b^ Jing-Jing Zhang,^b^ Xia Liu,^b^ Li-Lan Deng,^a^* Xiao-Ling Shen,*^c^*,* and Gang Xu*^b^,**

**Addresses:** ^a^ Southwest forestry university, Kunming 650224, P. R. China

^b^ State Key Laboratory of Phytochemistry and Plant Resources in West China, Kunming Institute of Botany, Chinese Academy of Sciences, Kunming 650204, P. R. China,

^c^ Laboratory of Chinese Herbal Drug Discovery, Tropical Medicine Institute, Guangzhou University of Chinese Medicine, Guangzhou 510405, P. R. China

**Corresponding author contact details:** Tel.: +86-871-65217971; e-mail: [xugang008@mail.kib.ac.cn](mailto:xugang008@mail.kib.ac.cn) (G. Xu), [xlshen66@126.com](mailto:xlshen66@126.com) (X.L. Shen).

**Contents:**

**S2** ^1^H NMR spectrum for **1** in C_5_D_5_N

**S3** ^13^C NMR and DEPT spectra for **1** in C_5_D_5_N

**S4** HSQC spectrum for **1** in C_5_D_5_N

**S5** ^1^H-^1^H COSY spectrum for **1** in C_5_D_5_N

**S6** HMBC spectrum for **1** in C_5_D_5_N

**S7** ROESY spectrum for **1** in C_5_D_5_N

**S8** IR spectrum for **1** (KBr)

**S9** HRTOFMS spectrum and data for **1**

**S2** ^1^H NMR spectrum for **1** in C_5_D_5_N


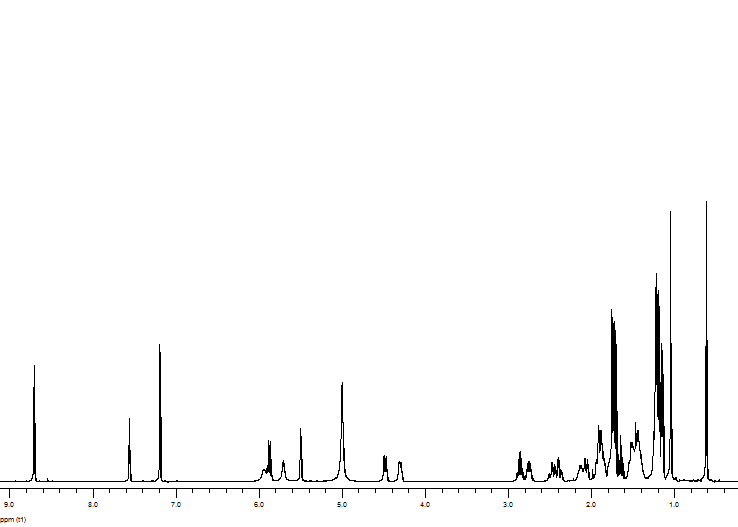


**S3** ^13^C NMR and DEPT spectra for **1** in C_5_D_5_N


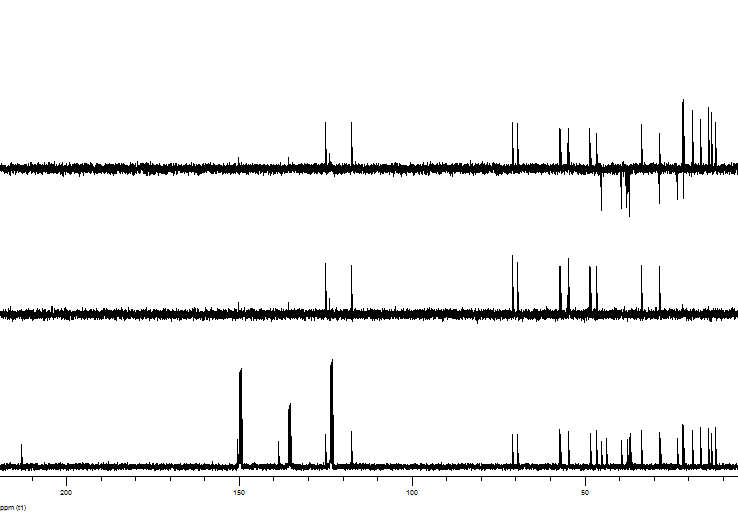


**S4** HSQC spectrum for **1** in C_5_D_5_N

**
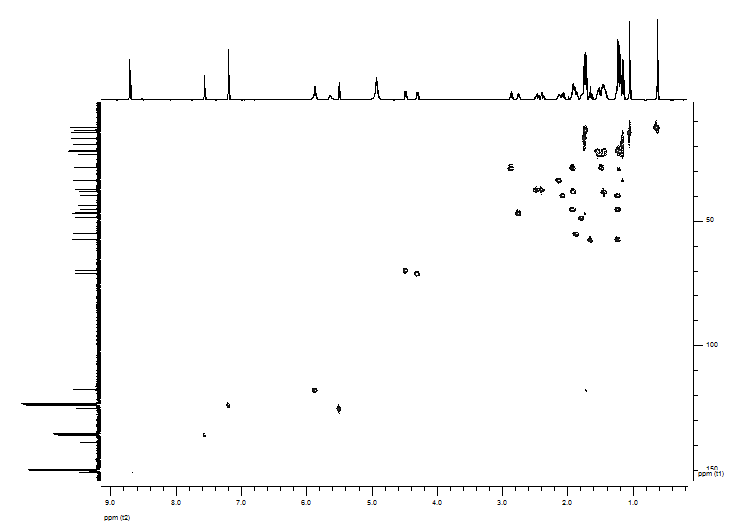
**

**S5** ^1^H-^1^H COSY spectrum for **1** in C_5_D_5_N

**
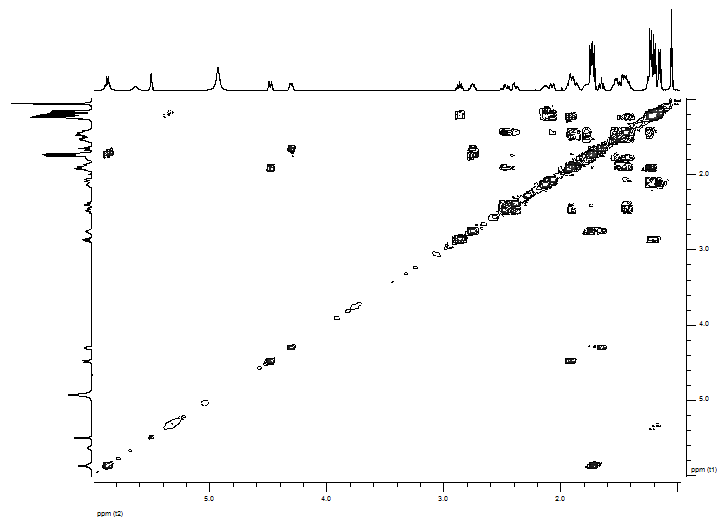
**

**S6** HMBC spectrum for **1** in C_5_D_5_N


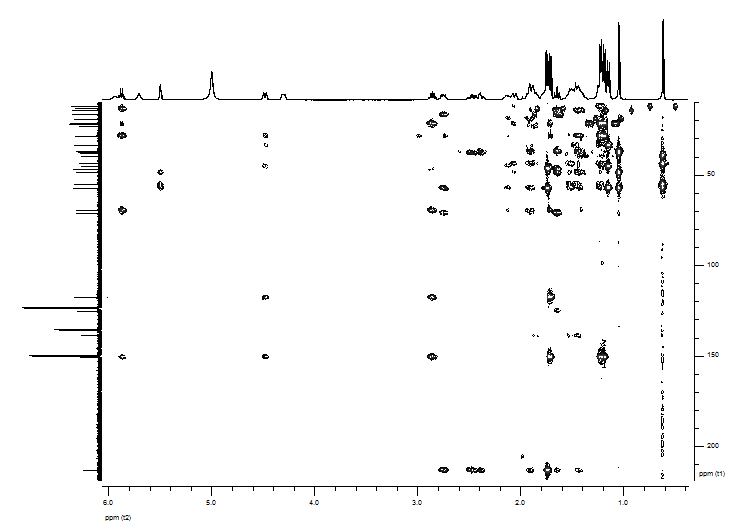


**S7** ROESY spectrum for **1** in C_5_D_5_N

**
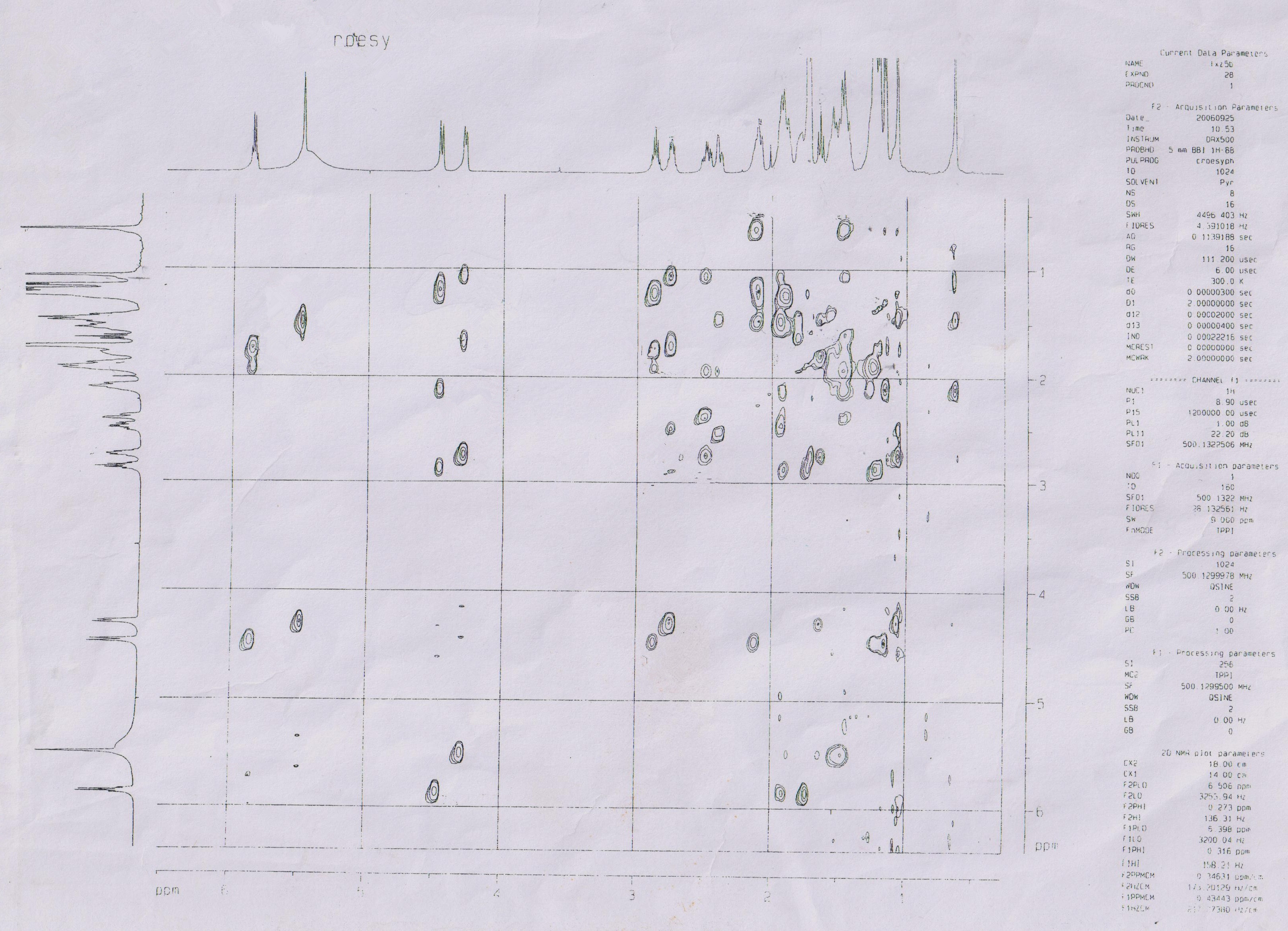
**

**S8** IR spectrum for **1** (KBr)


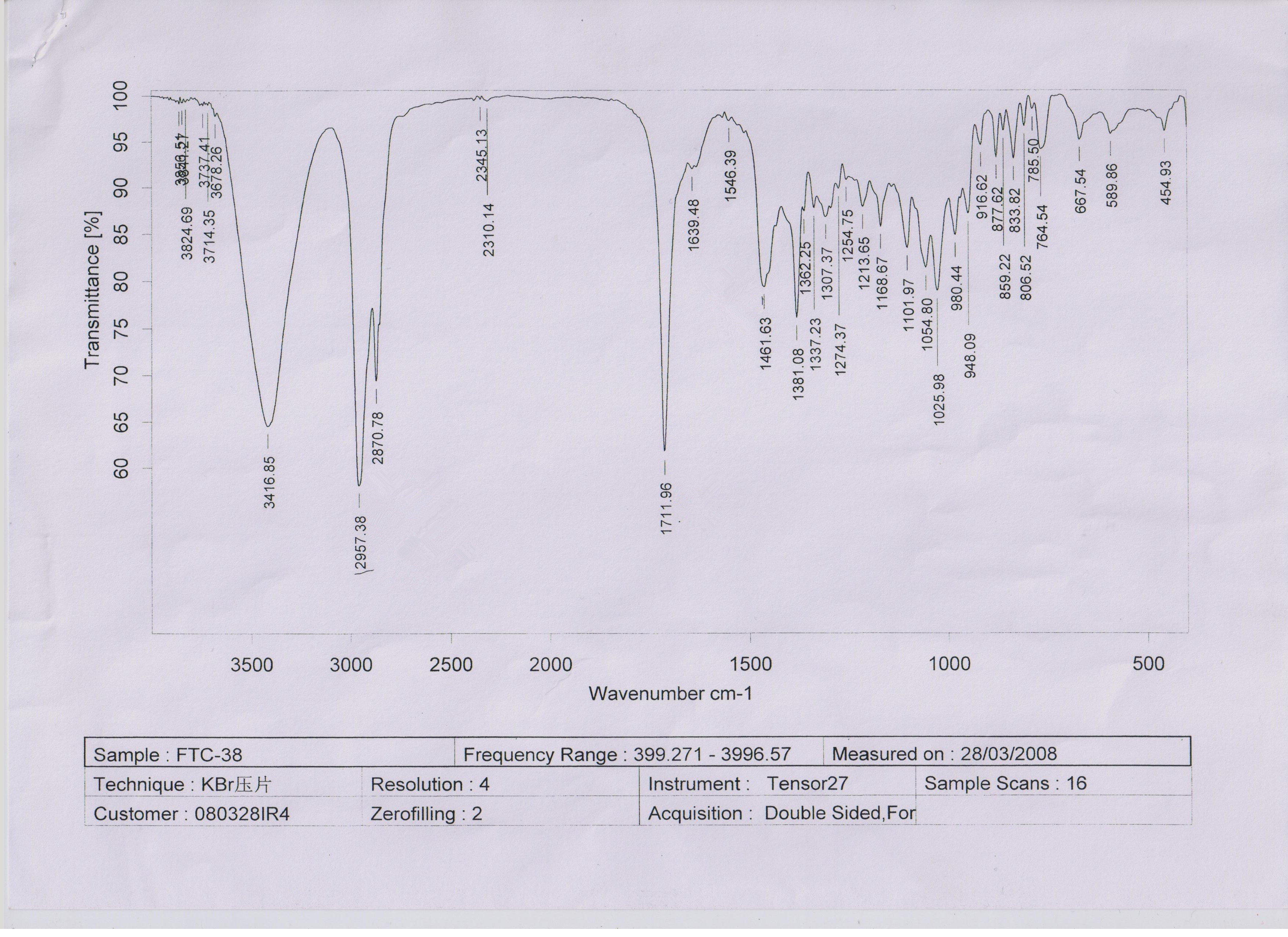


**S9** HRTOFMS spectrum and data for **1**


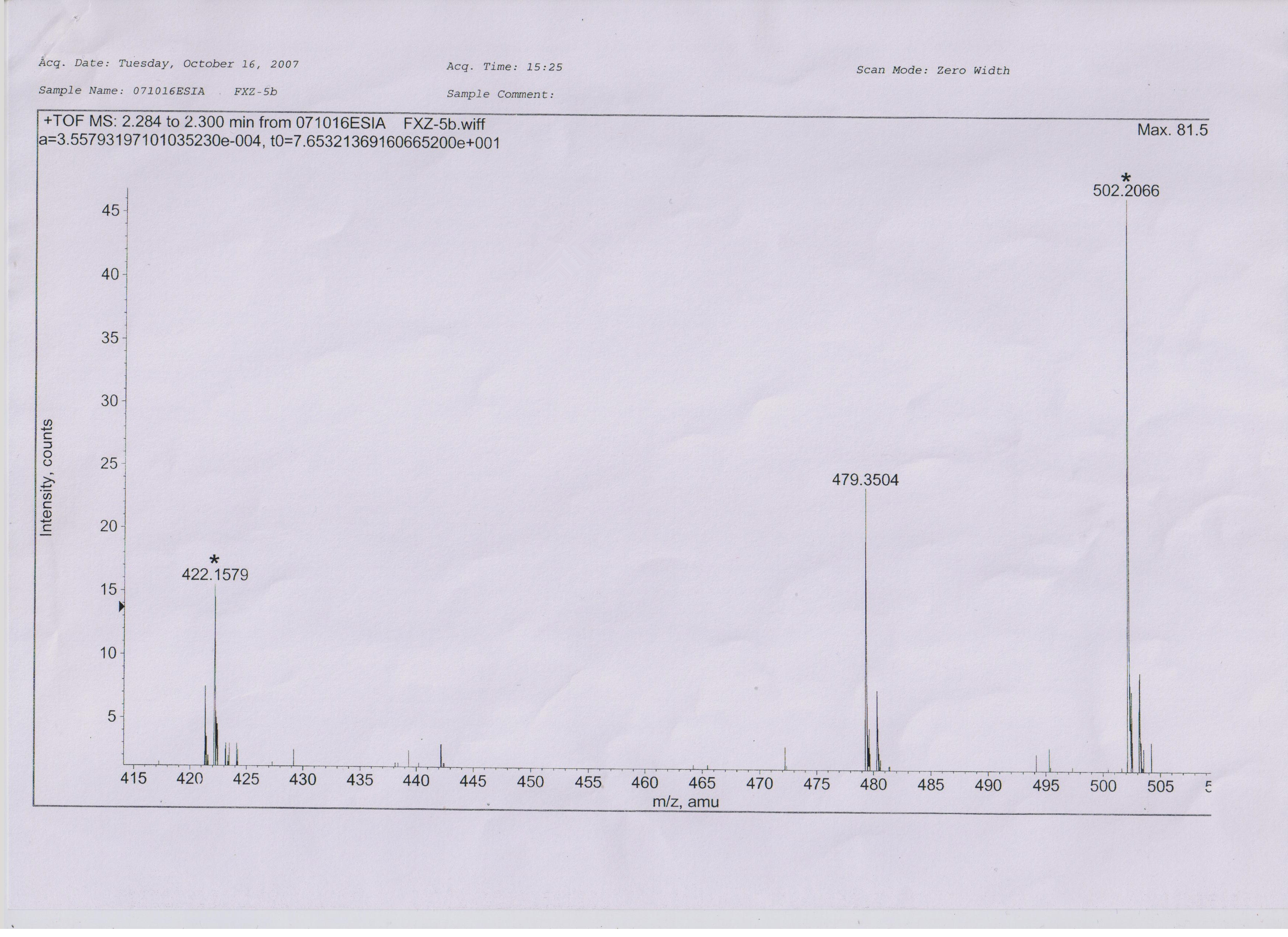


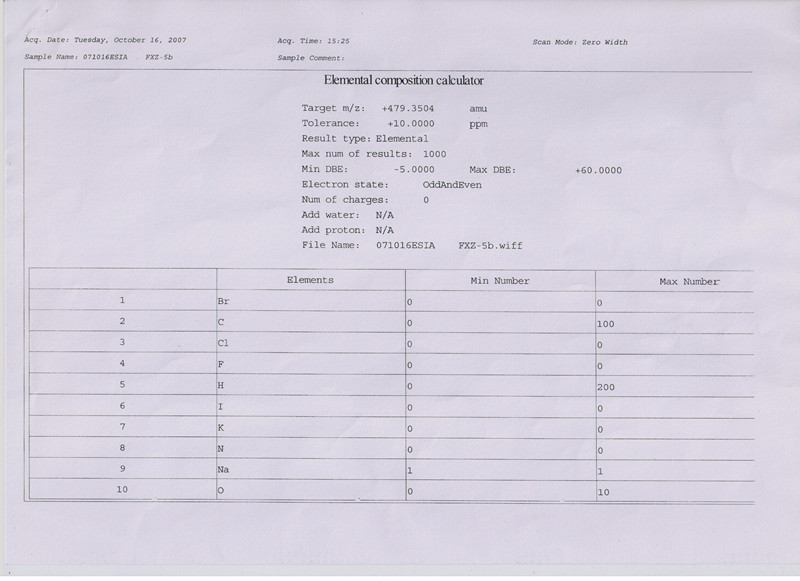


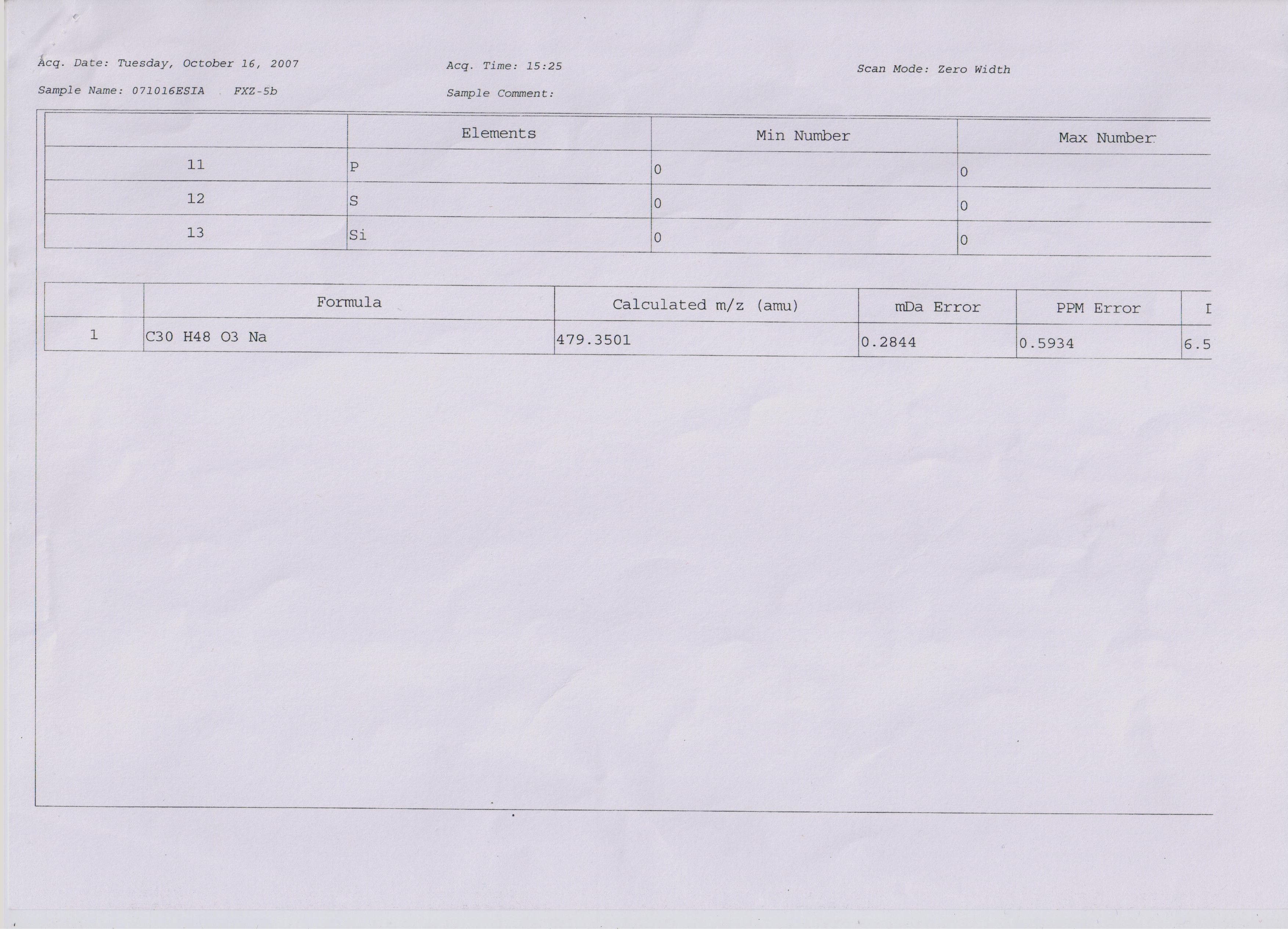

Supplement: Supplementary file 1 — Supplementary material 1 (DOC 5871 kb) [file 13659_2014_21_MOESM1_ESM.doc]
